# The Toronto Extremity Salvage Score in Unoperated Controls: An Age, Gender, and Country Comparison

**DOI:** 10.1155/2012/717213

**Published:** 2012-09-13

**Authors:** Mark Clayer, Simon Doyle, Nicole Sangha, Robert Grimer

**Affiliations:** ^1^Department of Orthopaedics, Royal Adelaide Hospital, North Terrace, Adelaide, SA 5000, Australia; ^2^University of Birmingham, Birmingham B15 2TT, UK; ^3^FRCS Royal Orthopaedic Hospital, Birmingham B31 2AP, UK

## Abstract

The Toronto Extremity Salvage Score (TESS) is widely used for the functional assessment of patients following surgery for musculoskeletal tumours. The aim of this study was to determine if there are gender and/or age-specific changes, unrelated to surgery, that may influence this score and the appropriateness of the questions. The TESS for lower limb was carried out in two different countries to see if there was variation between them. There were no statistically significant differences between the scores obtained between the respondents from Australia or Britain either in total or between the corresponding age groups. There were statistically significant differences in the TESS obtained between age groups with a lower score at older age groups but there was no difference between the sexes. Patients in the age group 70+ were more likely to record activities as “not applicable” and also have a lower score. This study has shown that age is the major factor in determining the TESS in both an Australian and British populations of otherwise healthy people. As there were no differences between the two populations, it supports the TESS as an international scoring system. There may be also an argument for age-specific questions.

## 1. Introduction 

The function of patients after treatment is an important consideration in the management of tumors of the extremities. In this context, function has been conceptualized in various ways. Examiner-dependent clinical measures and patient-reported outcomes are the most common. Some authors have used clinical measures, such as range of motion and muscle strength [1, 2] or a combination of symptoms and mobility [3, 4]. Others have used patient-reported outcomes such as the sickness impact profile [5] and the Toronto Extremity Salvage Score (TESS) [6, 7]. The TESS was based on the definitions of disability, impairment, and handicap as documented by the World Health Organization [8]. The TESS has been used for patients after surgery for extremity sarcoma [7, 9–11], and has been tested for validity and reliability (6,12) but a standard of what constitutes “normal” has not been undertaken. The questions then arise: does age make a difference to the score; does gender make a difference to the score and does cultural background affect the score achieved? These questions may be relevant as sarcoma classically affects specific ages; bone sarcomas generally affect those in the first and second decades of life whereas soft tissue sarcomas more commonly affect those in the sixth and seventh decades of life. What then constitutes a normal TESS score?

This study was developed to answer these questions and also to assess whether the questions asked are appropriate across ages and genders.

## 2. Patients and Methods 

The TESS is used routinely as an assessment tool in both Investigators' practices. The participants for this study were selected from the relatives/spouses of attendees at these practices. Inclusion and exclusion criteria were first developed using the criteria described by Davis et al. (1996) [6]. In brief, patients were considered eligible for inclusion if (1) they were between the ages of 30 and 79 years, (2) they had no known musculoskeletal disease or undergone joint replacement of the lower limb, and (3) they were able to read and write English. Patients were excluded if (1) they refused to consent to participate, (2) they had known cognitive impairments, or (3) they failed the above inclusion criteria. In addition, study participants were selected such that they were in the age groups of 30–39 years, 40–49 years, 50–59 years, 60–69 years, and 70–79 years. The TESS was administered in a paper format in the Investigators' clinics. The scores were then calculated using an Excel program (Microsoft Corp, Redmond, WA, USA). Statistical analysis of the demographic data was done using nominal and continuous reporting. Comparisons were then analyzed for Internet usage against the demographic data using Student's *t* test (Prism 5 software, GraphPad Software). 

There were 192 participants (100 Australian and 92 British). There were 89 men and 103 women. 

The compliance rate and completion rate were recorded in addition to ambiguous answers (i.e., when more than one response is given for a single question). In the case of an ambiguous answer, the answer was ranked down for scoring to the lower of the two responses. 

## 3. Results 

The scores were compared in total and then separately by age groups and gender between those recorded by Australian respondents and British respondents. There were no statistically significant differences between the cumulative scores or corresponding groups individually (Table 1). Therefore, all subsequent analyses for age and gender were done with the two groups combined. 

### 3.1. Age

The main question of this study was to determine if age made a significant difference to the scores obtained. In both populations, age did have an impact on the scores obtained. The TESS decreased with increasing age (Figure 2). In women the TESS dropped steadily from age 40 years with an unexplained rise in the 50–59 year old age group. The age group 30–39 years had statistically better TESS than all other age groups except women aged 50–59 years (Table 2). In men the TESS score remained relatively constant until age 70. There were no statistically significant differences between the male age groups except the age group of 30–39 years compared to the age group 60–69 years (*P* = 0.006) and all male groups had significantly higher TESS compared to the 70–79 year old male group (Table 2).

### 3.2. Gender

**Figure 1:**
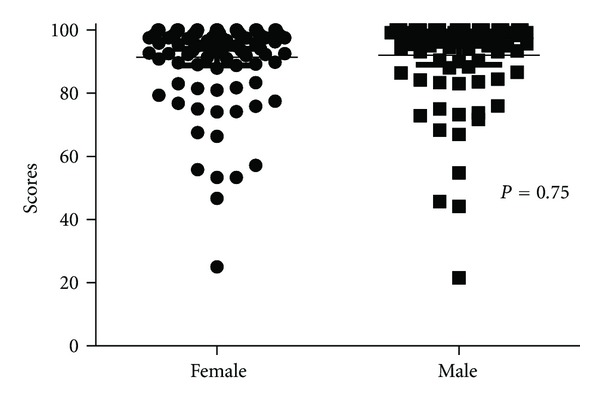
The TESS by gender (all patients).

**Figure 2:**
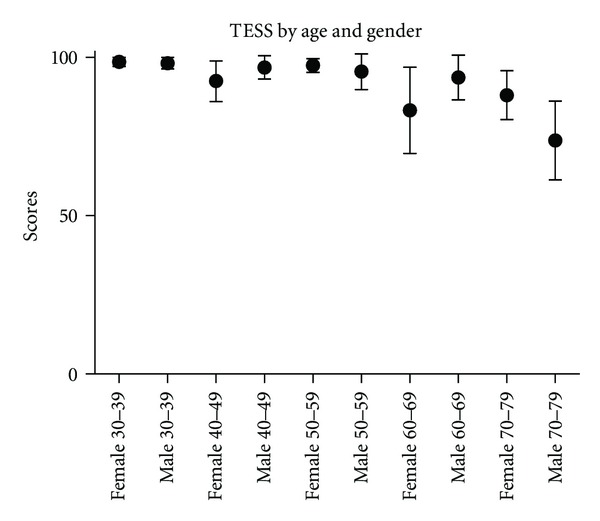
The TESS by age and gender (all patients).

There was no difference in scores between the genders, the mean TESS for all women was 91 which compared to 92 for men (*P* = 0.7) (see Figure 1). There were no gender-specific differences in the rating of the activities. It is interesting to note that there was minimal difference in the incidence of reporting difficulties with kneeling with 32 of the 153 females reporting difficulty with kneeling which compared to 30 males. Interestingly, 25 of the 50 Australian women reported difficulty with kneeling which compared to 7 British women and 24 Australian men reported difficulty with kneeling which compared to 6 British men. Similarly, 53 females reported difficulty getting up from kneeling which compared to 39 males (25 Australian women and 23 British, 25 Australian men and 14 British, resp.).

### 3.3. Country

There was no overall difference between the scores in Australia and England either at any age or between the sexes. As previously mentioned, the Australian respondents reported difficulty with kneeling more commonly than their British counterparts. This may be a cultural peculiarity as the British are more accustomed to kneeling before their royalty!

### 3.4. Applicability

The next question of the study was to determine if the questions asked were applicable for age and gender. The most common questions not felt to be relevant were those relating to sporting activities (37 respondents reported this as a “not applicable” question), followed by sexual activity (28 respondents) and working their usual number of hours (27 respondents) (Table 3). Twenty-five of those that answered that sport was not applicable for them were 60 years or older. Similarly, for sexual activity, 20 were 60 years or older and 24 out of the 27 that reporting that working their usual number of hours was not applicable were 60 years or older.

### 3.5. Compliance and Completion

All respondents approached agreed to participate, the compliance rate was 100%. There were 19 questions not answered (by 4 respondents) out of a possible 5790 questions, completion rate of 99.7% or, conversely, 188 questionnaires were completed out of a possible 192, completion rate of 97.9%.

Of the scores that were completed, the lowest score was for getting up from kneeling (mean 4.3 out of a maximum possible of 5), heavy housework (mean 4.4), kneeling (mean 4.5), walking up hills (mean 4.5), gardening and yard work (mean 4.5) and participating in usual sport (mean 4.5). In those who were 60 years or older, the greatest difficulty was with getting up from kneeling (mean 3.8), heavy housework (mean 4.1), kneeling (mean 4.1) and walking up hills (mean 4.1).

## 4. Discussion 

In the past, quality of life and assessments of function have relied on generic questionnaires such as the Short Form-36 ([12] or the Reintegration into Normal Living Index [13]. More thorough functional assessments after surgical procedures are now a mandatory requirement for the assessment of that procedure. The TESS has become the gold standard assessment tool for function after limb salvage surgery. Surgeon-reported outcomes are subject to bias and patient-reported outcomes have had variable success owing to bias, compliance, and completion. Assessments have a maximum score but it is not known if this maximum is achievable. Are there any factors, other than the condition that is being investigated, that may affect achievement of that maximum score? This study has investigated the factors of gender and age on an otherwise healthy population to determine if these do have an influence on achieving a maximum score. It has demonstrated that age is the most important factor that determines the TESS.

Our study is subject to several limitations. The first was that the number of participants was small. There are only 10 patients in each group in each country and so a full cross section of the population is not represented. The fact that the participants are related in some way to an individual with a musculoskeletal impairment may also influence their answers as well. It has also not taken into account other factors that have been shown to affect functional assessment such as obesity [14]. The study group does not reflect all cultures, and it may be argued that Great Britain and Australia do not have major cultural differences despite over 200 years of separation and a truly Asian population would be a more appropriate comparison group for cultural influences. Despite this, it has shown a cross-section of a population and confirmed that age does influence the TESS but not gender. This is important because the TESS is often used as a functional assessment tool for patients following Musculoskeletal tumour resection. To do this, it is important to have a “normal control” to compare the functional achievement obtained following surgery. Musculoskeletal tumours can affect any age but soft tissue sarcomas are more likely to affect those in the fifth or higher decade and bone sarcomas are most likely to affect those in the first two decades of life. This study has shown that the functional results obtained are likely to be affected by the age of the patient, not just the procedure performed. It also gives a standard to what can be achieved in relation to age and gender.

Reassuringly the results were essentially identical in two different countries which lends support to the validity of the TESS score as a suitable international comparison. The two countries involved are of course quite industrialised and both studies were carried out in large cities. The relevance of the score to other societies may of course be very different although most of the questions would be universally relevant. 

The fact that even in the over 60s most questions were answered is also reassuring but the decreasing proportion of questions felt to be relevant in the over 70s is simply a reflection of the more sheltered life this population generally leads.

This study has confirmed the usefulness of the TESS score across a wide age range but has shown the natural fall off in scores with ageing. Any study presenting the results of TESS scores that includes a proportion of patients over the age of 60 needs to be aware of this.

## Figures and Tables

**Table 1:** Mean TESS at different age groups split by country and gender and statistical relevance.

Age groups	Aus versus UK Female	*P*	Aus versus UK Male	*P*
30–39	98.5/98.3	0.9	97.8/100.0	0.9
40–49	92.5/91.5	0.9	96.8/93.5	0.4
50–59	97.4/95.4	0.5	95.5/96.5	0.8
60–69	85.4/83.5	0.8	93.7/92.5	0.8
70–79	88.1/88.9	0.9	73.7/80.2	0.6

**Table:** (a)

Age groups	Female 40–49	Female 50–59	Female 60–69	Female 70–79	Mean TESS
Female 30–39	0.03	0.2	0.001	0.001	98.4
Female 40–49		0.2	0.2	0.4	91.9
Female 50–59			0.01	0.02	96.5
Female 60–69				0.4	86.6
Female 70–79					88.4

**Table:** (b)

Age groups	Male 40–49	Male 50–59	Male 60–69	Male 70–79	Mean TESS
Male 30–39	0.06	0.2	0.006	0.0002	98.9
Male 40–49		0.8	0.4	0.003	95.4
Male 50–59			0.3	0.001	96.0
Male 60–69				0.006	93.1
Male 70–79					76.4

**Table 3:** Applicability of the questions in the TESS by country. Most common questions listed as “not applicable” by respondents. Results given as the total questions answered as “not applicable”/total number of respondents.

	Total number	UK	Australia
Participating in sporting activities	37/192	14/92	23/100
Participating in sexual activities	28/192	17/92	11/100
Working my usual number of hours	27/192	15/92	12/100
Completing my usual duties at work	19/192	14/92	5/100
Driving	19/192	14/92	5/100
Getting in and out of a bath	10/192	3/92	7/100

## References

[1] Lampert MH, Gerber LH, Glatstein E (1984). Soft tissue sarcoma: functional outcome after wide local excision and radiation therapy. *Archives of Physical Medicine and Rehabilitation*.

[2] Robinson MH, Spruce L, Eeles R (1991). Limb function following conservation treatment of adult soft tissue sarcoma. *European Journal of Cancer*.

[3] Enneking W, Enneking W (1987). Modification of the system for functional evaluation in the surgical management of musculoskeletal tumours. *Limb Salvage in Musculoskeletal Oncology*.

[4] Enneking WF, Dunham W, Gebhardt MC, Malawar M, Pritchard DJ (1993). A system for the functional evaluation of reconstructive procedures after surgical treatment of tumors of the musculoskeletal system. *Clinical Orthopaedics and Related Research*.

[5] Sugarbaker PH, Barofsky I, Rosenberg SA, Gianola FJ (1982). Quality of life assessment of patients in extremity sarcoma clinical trials. *Surgery*.

[6] Davis AM, Wright JG, Williams JI, Bombardier C, Griffin A, Bell RS (1996). Development of a measure of physical function for patients with bone and soft tissue sarcoma. *Quality of Life Research*.

[7] Davis AM, Bell RS, Badley EM, Yoshida K, Williams JI (1999). Evaluating functional outcome in patients with lower extremity sarcoma. *Clinical Orthopaedics and Related Research*.

[8] World Health Organization (1990). *International Classification of Impairments, Disabilities and Handicaps*.

[9] Davis AM, Sennik S, Griffin AM (2000). Predictors of functional outcomes following limb salvage surgery for lower-extremity soft tissue sarcoma. *Journal of Surgical Oncology*.

[10] Gerrand CH, Wunder JS, Kandel RA (2004). The influence of anatomic location on functional outcome in lower-extremity soft-tissue sarcoma. *Annals of Surgical Oncology*.

[11] Schreiber D, Bell RS, Wunder JS (2006). Evaluating function and health related quality of life in patients treated for extremity soft tissue sarcoma. *Quality of Life Research*.

[12] Thijssens KMJ, Hoekstra-Weebers JEHM, Van Ginkel RJ, Hoekstra HJ (2006). Quality of life after hyperthermic isolated limb perfusion for locally advanced extremity soft tissue sarcoma. *Annals of Surgical Oncology*.

[13] Nagarajan R, Mogil R, Neglia JP, Robison LL, Ness KK (2009). Self-reported global function among adult survivors of childhood lower-extremity bone tumors: a report from the Childhood Cancer Survivor Study (CCSS). *Journal of Cancer Survivorship*.

[14] Kortt MA, Dollery B (2011). Association between body mass index and health-related quality of life among an Australian sample. *Clinical Therapeutics*.

